# *Edwardsiella tarda* in Tambaqui (*Colossoma macropomum*): A Pathogenicity, Antimicrobial Susceptibility, and Genetic Analysis of Brazilian Isolates

**DOI:** 10.3390/ani13182910

**Published:** 2023-09-14

**Authors:** Francisco Yan Tavares Reis, Victória Pontes Rocha, Peter Charrie Janampa-Sarmiento, Henrique Lopes Costa, Renata Catão Egger, Naísa Cristine Passos, Carlos Henrique Santos de Assis, Sarah Portes Carneiro, Ágna Ferreira Santos, Brendhal Almeida Silva, Fernanda Alves Dorella, Márcia Pimenta Leibowitz, Ronald Kennedy Luz, Felipe Pierezan, Sílvia Umeda Gallani, Guilherme Campos Tavares, Henrique César Pereira Figueiredo

**Affiliations:** 1 Department of Preventive Veterinary Medicine, School of Veterinary Medicine, Federal University of Minas Gerais, Belo Horizonte 31270-901, MG, Brazil; yan_reis@hotmail.com (F.Y.T.R.); mvvictoriapr@gmail.com (V.P.R.);; 2 Department of Veterinary Clinics and Surgery, School of Veterinary Medicine, Federal University of Minas Gerais, Belo Horizonte 31270-901, MG, Brazil; 3 Department of Animal Science, School of Veterinary Medicine, Federal University of Minas Gerais, Belo Horizonte 31270-901, MG, Brazil; 4 Postgraduate Program in Aquaculture, Nilton Lins University, Manaus 69058-030, AM, Brazil

**Keywords:** amazon fish, subclinical, edwardsiellosis, Koch’s postulate, genetic typing

## Abstract

**Simple Summary:**

Edwardsiellosis is a disease caused by bacteria of the genus *Edwardsiella*, like *Edwardsiella* tarda, which mainly affects fish. In Brazil, this bacterium has been found in many tambaquis since 2014; however, an investigation into how this fish is affected and the potential implications of this has never been conducted. After experimental infection, we found that tambaqui seem clinically healthy; however, their internal organs were replete with histological lesions called granulomas. The bacteria found could also be grouped into three distinct genetic groups and had no reduced susceptibility to florfenicol, norfloxacin, neomycin, erythromycin, or oxytetracycline. This study raises awareness of the occurrence of *E. tarda* causing subclinical histological lesions in tambaqui, a highly important fish for the Brazilian aquaculture.

**Abstract:**

*Edwardsiella tarda* is a crucial pathogenic bacterium in tropical aquaculture. This bacterium was recently isolated from tambaqui (*Colossoma macropomum*), a commercially important fish species in Brazil. This study assessed the antimicrobial susceptibility, pathogenicity, and genetic diversity of the tambaqui-derived *E. tarda* isolates. Fourteen bacterial isolates isolated from tambaqui were identified as *E. tarda* by using matrix-assisted laser desorption/ionization–time-of-flight mass spectrometry and *dnaJ* gene sequencing. Antimicrobial susceptibility tests were conducted against seven drugs using the disc diffusion assay. The pathogenicity test conducted by intraperitoneal injection of 2.4 × 10^7^ colony-forming units (CFU) fish^−1^ of *E. tarda* (ED38-17) into tambaqui juveniles eventually revealed that neither clinical signs nor death were present. However, splenomegaly and whitish areas in the spleen and kidneys were observed. The histological investigation also revealed granulomatous splenitis, nephritis, and hepatitis occurring internally. Repetitive extragenic palindromic-PCR fingerprinting separated the 14 isolates into three genetic groups. The antibiogram revealed that all *E. tarda* isolates were wild-type (WT) to florfenicol (FLO), norfloxacin (NOR), neomycin (NEO), erythromycin (ERY), and oxytetracycline (OXY); however, some were non-wild-type to sulfamethoxazole/trimethoprim (7.1%) and amoxicillin (21.4%). Therefore, through experimental infection, *E. tarda* ED38-17 could induce pathogenic effects in *C. macropomum*. Additionally, three distinct genetic types were found, and the *E. tarda* isolates were WT to FLO, NOR, NEO, ERY, and OXY. These findings raise awareness of a bacteria causing unseen lesions, a pathogen that will potentially impact tambaqui aquaculture in the future.

## 1. Introduction

Tambaqui (*Colossoma macropomum*) is an economically important freshwater Amazon fish species. This species is cultured for human consumption in many South American countries, such as Colombia, Peru, Venezuela, Bolivia, Ecuador, and especially in Brazil [1]. Brazilian production reached 101.1 thousand tons in 2019, making tambaqui the second-most-produced fish species in Brazil [2]. A substantial part of production is intended for exportation, with a total of 225 tons having been exported in 2021 [3]. 

Sanitary issues that occur during the production of this species are largely caused by parasites, such as *Neoechinorhynchus buttnerae* [4]. Bacterial infections are also observed, but at a smaller scale. *Aeromonas hydrophila, Aeromonas jandaei* and *Flavobacterium columnare* are actually the few known bacterial species scientifically established to be pathogens of *C. macropomum* [5,6]. 

*Edwardsiella tarda* is a Gram-negative, facultative anaerobic, rod-shaped bacterium belonging to the *Hafniaceae* family. Along with *E. tarda*, four other species belonging to the *Edwardsiella* genus have been found: *E. hoshinae*, *E. ictaluri*, *E. piscicida*, and *E. anguillarum*. *E. piscicida* and *E. anguillarum* had been misclassified as *E. tarda* for a long time, since phenotypic differences could not be observed between these species [7,8]. Many methods can be used to distinguish these species, such as matrix-assisted laser desorption/ionization–time-of-flight (MALDI-TOF), repetitive extragenic palindromic-PCR (REP-PCR), *gyrB* and *sodB* gene sequencing, and multiplex PCR [9]. *E. tarda* is known to infect fish, leading to high economic losses [10]; however, it has also been isolated from reptiles [11], birds [12], amphibians [13], and humans [14]. *E. tarda* has been previously isolated from *Myleus micans*, a fish closely related to *C. macropomum*; it leads to a high mortality rate in that species. Moreover, *E. tarda* showed pathogenicity against *Oreochromis* spp. and *Cyprinus carpio* after experimental infection [15]. Although *E. tarda* has been previously isolated from tambaqui [16], its pathogenicity against this fish has still not been investigated. 

*E. tarda* is susceptible to many antimicrobials; however, due to its facultative intracellular characteristics, higher doses are required to effectively treat *E. tarda*-infected fish compared to those required to treat infections caused by extracellular pathogens [17]. However, the inappropriate use of antimicrobials in aquaculture has led to the development of resistant bacteria. For instance, Xiao et al. [18] isolated a highly virulent isolate of *E. tarda* from farmed turbot (*Scophthalmus maximus*) that was non-wild-type (NWT) to chloramphenicol, tetracycline, streptomycin, and rifampicin. This biological hazard may pose a risk to humans if contaminated raw fish is consumed by humans, as it could potentially lead to persistent gastrointestinal infections [19,20]. 

This study aimed to investigate isolates of *E. tarda* found in Brazilian tambaqui farms during routine bacteriological examinations. This is the first report to associate *E. tarda* as cause of granulomatous lesions in tambaqui without clinical signs. Three distinct genetic groups were within the *E. tarda* isolates recovered from tambaqui and the antimicrobial susceptibility of these isolates was determined.

## 2. Materials and Methods

### 2.1. E. tarda Isolate Collection

During routine bacteriological examinations performed on cultured tambaqui in the Laboratory of Aquatic Animal Diseases (Aquavet-Veterinary School, Federal University of Minas Gerais, Belo Horizonte, Brazil) and the Laboratory of Applied Microbiology of Aquatic Organisms (LAMAO, Nilton Lins University, Manaus, Brazil), 14 isolates of *E. tarda* were found. These isolates were isolated from Brazilian tambaqui facilities located in Amazonas, Rondônia, and Minas Gerais between 2014 and 2020 (Table 1). Some of these facilities were suffering from fish mortality, which was accompanied by clinical signs of lethargy, melanosis, gill necrosis, and fin and skin erosions.

### 2.2. Bacteriological Examinations

Bacteriological examination was performed immediately upon the arrival of the fish at the laboratory. First, the fish were euthanized via immersion in 450 mg L^−1^ benzocaine solution (Sigma-Aldrich, Saint Louis, MO, USA), and any clinical signs of diseases were recorded. The fish were necropsied and swabs of the brain, kidney, liver, intestines, and spleen were aseptically collected, streaked onto brain heart infusion (BHI) agar (KASVI, Pinhais, Brazil) or tryptic soy agar (TSA) (HiMedia, Mumbai, India), and incubated at 28 °C for 72 h to isolate the bacterial pathogens. Thereafter, the isolates were subjected to Gram staining and streaked onto fresh TSA (HiMedia) or BHI (KASVI) agar. After bacterial growth, identification was conducted, and the isolates were stored in tryptic soy broth containing 15% glycerol at −80 °C until use.

### 2.3. Edwardsiella Tarda Identification

#### 2.3.1. MALDI-TOF MS Real-Time Identification

Real-time identification of the bacterial species using matrix-assisted laser desorption/ionization–time-of-flight mass spectrometry (MALDI-TOF MS) was conducted in the following manner. A single CFU was collected and applied onto one of the 96 spots of a stainless-steel target plate (Bruker Daltonics, Billerica, MA, USA) using a sterile wooden toothpick. Then, 1 µL of 70% formic acid (Sigma-Aldrich) was added and the mixture was air-dried, after which 1 µL of an α-cyano-4-hydroxycinnamic acid (HCCA) matrix (Bruker Daltonics) was applied to the spot and allowed to dry naturally. This process was performed for all of the 14 *E. tarda* isolates. Spectra were acquired using FlexControl software version 3.4 and a Microflex LT mass spectrometer (Bruker Daltonics), while following the manufacturer’s recommendations. The m/z value range was 1960-20137 Da and the electrical voltages of ion sources 1 and 2 were 19.99 and 18.24 kV, respectively, while the electrical voltage of the lens was 6.0 kV. The bacterial test standard (*E. coli* DH5 alpha; Bruker Daltonics) was used to calibrate the device. Scores ≥ 2000 indicated species identification.

Additionally, species-specific peptide mass peaks unique to *E. tarda* (m/z = 4252), *E. piscicida* (m/z = 8793), and *E. anguillarum* (m/z = 7628) were searched by analyzing the spectra obtained using FlexAnalysis software version 3.4 (Bruker Daltonics), as recommended by Reichley et al. (2017).

#### 2.3.2. Molecular Confirmation of Identification

All of the selected isolates were thawed, streaked onto MacConkey agar (HiMedia, Mumbai, India), and incubated at 28 °C for 24 h. A Maxwell 16 Tissue DNA purification kit (Promega, Madison, WI, USA) was used to extract the bacterial DNA as recommended by the manufacturer. Then, a Nanodrop spectrophotometer (Thermo Scientific, Wilmington, NC, USA) was used to quantify the extracted DNA and was further stored at −20 °C until use.

To confirm the results obtained for the identification of all 14 *E. tarda* isolates at the species level using MALDI-TOF, *dnaJ* gene sequencing was conducted, as previously described by [21], with some modifications. The amplification reaction was carried out using the primers DN1-1F (5′-GATYTRCGHTAYAACATGGA-3′) and DN1-2R (5′-TTCACRCCRTYDAAGAARC-3′). Primers were synthesized and purified by Integrated DNA Technologies (Coralville, IA, USA). PCR was executed using a Hot Start Taq polymerase kit (Qiagen, Valencia, CA, USA) in a final reaction volume of 25 µL. The reaction mixture was composed of 1× PCR buffer, 0.2 mM dNTPs, 2 mM MgCl_2_, 0.25 mM of each primer, 1.5 U of Taq DNA polymerase, and 100 ng of template DNA. The PCR conditions were as follows: an initial step at 94 °C for 15 min, followed by 40 cycles at 94 °C for 45 s, 51 °C for 45 s, 72 °C for 1 min, and a final elongation at 72 °C for 5 min. Amplification was performed using a Veriti 96-well thermal cycler (Life Technologies, Thermo Scientific, USA), and the PCR amplicons were separated using a QIAxcell Advanced and a QX DNA Screening Kit (Qiagen).

PCR amplicons were purified using Agencourt AMPure XP (Beckman Coulter, Pasadena, CA, USA) as recommended by the manufacturer. A BigDye^TM^ Terminator Cycle Sequencing kit (Applied Biosystems, Carlsbad, CA, USA), combined with the primers used for the *dnaJ* PCR procedure, were used to amplify the sequences, which were then evaluated using an ABI 3500 Genetic Analyzer (Life Technologies, Carlsbad, CA, USA). The sequencing products, forward and reverse, were merged using the BioEdit software (Ibis BioSciences, Carlsbad, CA, USA) version 7.2, resulting in contigs. The *National Center for Biotechnology Information* (NCBI) database was then searched for sequences with high similarity to the generated contigs using the BLAST web server [22].

Also, a phylogenetic tree was constructed with the *dnaJ* gene sequences of the 14 isolates recovered from the tambaqui and *dnaJ* gene sequences (719 bp) of *E. tarda* (NZ_CP023706.1; AB454434.1), *E. hoshinae* (AB272631.1), *E. ictaluri* (NC_012779.2), *E. anguillarum* (CP095163.1), *E. piscicida* (QCZQ01000004.1), and *Serratia rubidaea* (LJZP01000034.1), retrieved from NCBI database. MEGA software version 11 [23] was employed to construct the tree. The alignment of sequences was performed with the ClustalW algorithm and the neighbor-joining method with the Jukes–Cantor model was used to construct a tree with 1000 bootstrap pseudoreplicates.

### 2.4. Genetic Typing Using Repetitive Extragenic Palindromic-PCR (rep-PCR)

To evaluate the genetic diversity of the *E. tarda* isolates, rep-PCR was performed as previously described by Costa et al. [24]. The amplification reaction was performed using a GTG_5_ primer (5′-GTGGTGGTGGTGGTG-3′) (Life Technologies, Thermo Scientific, Waltham, MA, USA). PCR was performed using a HotStart Taq polymerase kit (Qiagen) in a final reaction volume of 25 µL. The reaction mixture was composed of 1× PCR buffer, 0.2 µM dNTPs, 1.5 mM MgCl_2_, 0.5 µM GTG_5_ primer, 2 U Taq DNA polymerase, and 35 ng of template DNA. The PCR process was carried out using a Veriti 96-well thermal cycler (Applied Biosystems, Waltham, MA, USA). The PCR conditions were as follows: an initial step at 95 °C for 15 min, followed by 30 cycles at 95 °C for 30 s, 45 °C for 1 min, and 72 °C for 4 min. Finally, a final elongation step was performed at 72 °C for 16 min.

The products of the PCR were dissociated via electrophoresis using a 1.5% agarose gel and stained with ethidium bromide (0.5 µg mL^−1^ for 20 min). Ladders of 1 kb (Promega, USA) were used as the molecular size standards. PCR gels were visualized via UV transillumination, and images were captured using an L-Pix EX digital imaging system (Loccus Biotecnologia, Cotia, Brazil). Rep-PCR gel images were analyzed using BioNumerics version 6.6 (Applied Maths, Kortrijk, Belgium). The Dice coefficient was used to determine the similarities between banding patterns [25], and a dendrogram was created using the unweighted pair group method with the arithmetic mean (UPGMA) approach. To consider the isolates as clonally related, a cutoff based on Dice similarity was proposed, considering the mean value of all clusters minus 1645 per standard deviation (i.e., considering the normal distribution curve, 95% of the similarity values will be above the indicated cutoff). The discriminatory power of rep-PCR was determined using the Simpson diversity index [26].

### 2.5. Antimicrobial Susceptibility Testing 

The disk diffusion assay was conducted in accordance with the VET03 guidelines established by the Clinical and Laboratory Standard Institute [27]. Here, the aim was to address the variability of antimicrobial susceptibility of *C. macropomum*-derived *E. tarda* isolates; hence, antimicrobials from different antimicrobial classes were included. Commercially available disks (Oxoid, Basingstoke, UK) containing the antimicrobials florfenicol (FLO, 30 μg), norfloxacin (NOR, 10 μg), neomycin (NEO, 10 μg), erythromycin (ERY, 15 μg), trimethoprim-sulfamethoxazole (SXT, 25 μg), amoxicillin (AMO, 10 μg), and oxytetracycline (OXY, 30 μg) were obtained. However, quality control ranges for NOR, NEO, and AMO were not yet established by CLSI. All of the 14 selected *E. tarda* isolates were thawed, streaked onto MacConkey agar (HiMedia), and incubated at 28 °C for 24 h. Following incubation, each isolate was collected and suspended in sterile saline to achieve a 625 nm absorbance value in the 0.08–0.13 range, which was measured using a visible spectrophotometer (Spectrum, China). Muller–Hinton agar (Sigma-Aldrich) plates were inoculated with bacteria using sterile swabs. The antimicrobial disks were then placed on agar and the plates were incubated at 28 °C for 24 h. The analysis was performed in triplicate. Moreover, the *Escherichia coli* isolate ATCC 25922 and *Aeromonas salmonicida* subsp. *salmonicida* isolate ATCC 33658 (quality control isolates) were cultured on MacConkey agar (HiMedia) at 28 °C for 24 h. The experimental conditions for these strains were identical to those described above. The diameter of each inhibition zone was measured using a ruler and the averages of the triplicates were used for further analysis. The isolates were classified as either wild-type (WT) or non-wild-type (NWT), based on provisional epidemiological cutoff values (CO_WT_) calculated via normalized resistance interpretation (NRI) [28,29,30]. To meet the minimum requirements of the NRI method [31], the disc diffusion data of the *E. tarda* isolates isolated from *Oreochromis niloticus* (n = 2), *Arapaima gigas* (n = 2), *Brycon amazonicus* (n = 1), *Symphysodon* spp. (n = 1), and *Pterophyllum scalare* (n = 1) from the LAMAO culture collection were included in the calculation of the CO_WT_. NRI analysis was conducted using an online MS Excel spreadsheet program that was made available online by Smith, Finnegan, and Kronvall [32]. NWT isolates categorized thus for at least three antibiotics were classified as multiple-drug reduced-susceptibility bacteria [33].

### 2.6. Pathogenicity Evaluation

#### 2.6.1. Fish and Experimental Infection 

To estimate the pathogenic potential for tambaqui, fish were experimentally infected with a randomly selected *E. tarda* isolate, ED38-17. The Ethics Committee on Animal Use of the Federal University of Minas Gerais approved all procedures (protocol number 152/2020).

Eighteen *C*. *macropomum* juveniles without clinical signs and with an average body weight of 57.68 ± 17.95 g were acquired from the bioterium of the institution. Upon arrival, the fish were acclimated for 15 days in three glass aquaria containing 57 L of dechlorinated water, and half of this volume was renewed every two days. The water temperature was maintained at 28 °C and air stones were used to supply the appropriate level of dissolved oxygen. Commercial fish feed containing 32% protein (Socil, São Paulo, Brazil) was provided to tambaqui juveniles twice per day (3% of body weight day^−1^). 

Prior to infection, six fish were randomly selected and verified to be free of bacterial infection after bacteriological analysis of the aseptically collected brain, kidney, spleen, and liver swabs, which were streaked onto TSA and Hsu–Shotts agar (MHS) and then incubated at 28 °C for 48 h. Also, a bacterial solution of *E. tarda* (ED38-17) was prepared by adding the bacteria (grown on BHI agar at 28 °C for 24 h) to an Erlenmeyer containing BHI broth, allowing it to grow over six hours at 28 °C and 100 rpm. As subsequently described, two treatments were applied to two different groups: challenged and non-challenged. After 24 h of starvation, sedation of the animals was performed through immersion in a benzocaine solution (100 mg L^−1^). The fish in the challenged group were subjected to intraperitoneal injection of 0.1 mL of *E. tarda* (ED38-17) bacterial dose at 2.4 × 10^8^ CFU mL^−1^, while the fish in the non-challenged group were intraperitoneally injected with the same volume of sterile BHI broth (KASVI). Clinical signs and mortality were recorded four times per day for 21 days. During this period, the water and fish were managed as in the acclimation phase, as previously described. At the end of the challenge period, the surviving fish were euthanized (benzocaine bath; 300 mg L^−1^), necropsied, and subjected to bacteriological investigation. This was conducted by aseptically sampling the brain, kidney, spleen, and liver; each organ was divided in two segments, so that one of the segments remained intact for the histological analysis, and the other segment was streaked onto TSA and incubated at 28 °C for 48 h. Real-time identification using MALDI-TOF MS was performed as soon as bacterial colony growth was detected (see Section 2.3.). 

#### 2.6.2. Histological Analysis

A histological analysis was performed to evaluate the pathogenicity of *E. tarda* toward *C. macropomum* at the tissue level. Liver, posterior kidney, and spleen samples were collected from each fish at the end of the infection period and immersed in 10% buffered formalin for 24 h. The organs were dehydrated by immersion in increasing concentrations of ethanol (70–100%), cleared with xylene, and embedded in paraffin wax. Tissue sections (thickness: 4 µm) were obtained from each organ using a semi-automated rotary microtome Leica RM2245 (Leica Biosystems, Wetzlar, Germany) and stained with hematoxylin-eosin (HE) [34]. Sections were observed using a Leica DM4000 B microscope (Leica Biosystems, Nussloch, Germany) and photographed using a Leica DFC 500 digital camera (Leica Biosystems, Nussloch, Germany).

## 3. Results

### 3.1. Bacterial Identification 

MALDI-TOF MS analysis could identify all 14 isolates as *E. tarda* at the species level, with scores varying between 2153 and 2600 (Table 1). Moreover, the *E. tarda*-specific peptide peaks (m/z between 4249.280 and 4254.087) were observed for these isolates. *E. piscicida*- or *E. anguillarum*-specific peptide peaks were not detected.

**Figure 1:**
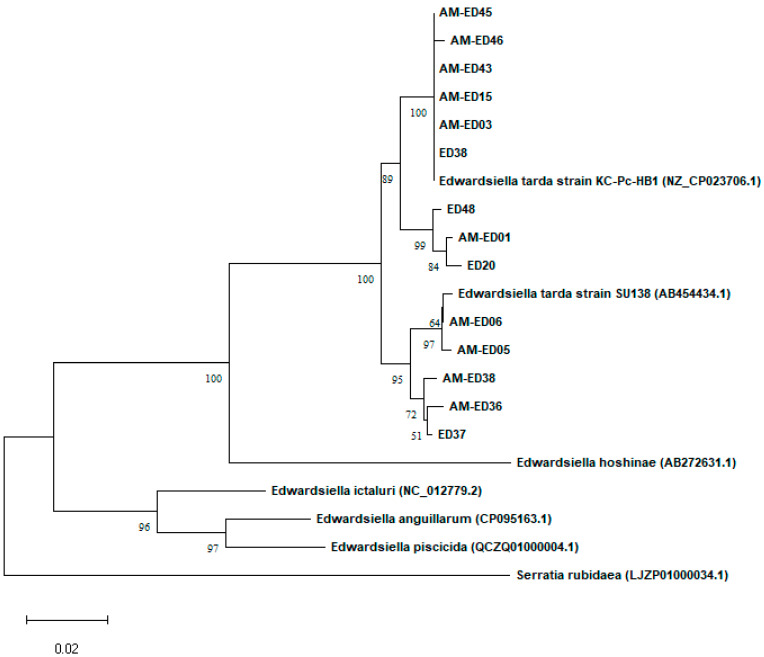
Phylogenetic tree constructed using the neighbor-joining method based on *dnaJ* gene sequences (719 bp) from *C. macropomum*-derived *E. tarda* isolates. Known *E. tarda* (NZ CP023706.1; AB454434.1), *E. hoshinae* (AB272631.1), *E. ictaluri* (NC 012779.2), *E. anguillarum* (CP095163.1), *E. piscicida* (NZ QCZQ1000004.1), and *Serratia rubidaea* (LJZP01000034.1) are also present. Numbers on the branches indicate bootstrap percentage after 1000 replications in constructing the tree. Scale bar refers to a phylogenetic distance of 14.38 nucleotide substitutions per site.

The PCR amplification of *dnaJ* yielded a product of approximately 729 bp. The sequences obtained from the 14 isolates had a query coverage of 100% and an identity ranging from 97.81% to 100% with other *E. tarda* sequences deposited in the NCBI database, according to the BLASTn analysis results. Tambaqui-derived *E. tarda* sequences were also added to NCBI database with the following accession numbers: OP535031 (AM-ED01); OP535032 (AM-ED03); OP535033 (AM-ED05); OP535034 (AM-ED06); OP535035 (AM-ED15); OP535036 (AM-ED36); OP535037 (AM-ED38); OP535038 (AM-ED43); OP535039 (AM-ED45); OP535040 (AM-ED46); OP535041 (ED20-14); OP535042 (ED37-17); OP535043 (ED38-17); and OP535044 (ED48-20). Ultimately, the phylogenetic tree showed that all 14 *dnaJ* gene sequences were placed within clusters where sequences of known *E. tarda* were placed (Figure 1), confirming the identification of the isolates as *E. tarda.*

### 3.2. Genetic Typing of the E. tarda Isolates

**Figure 2:**
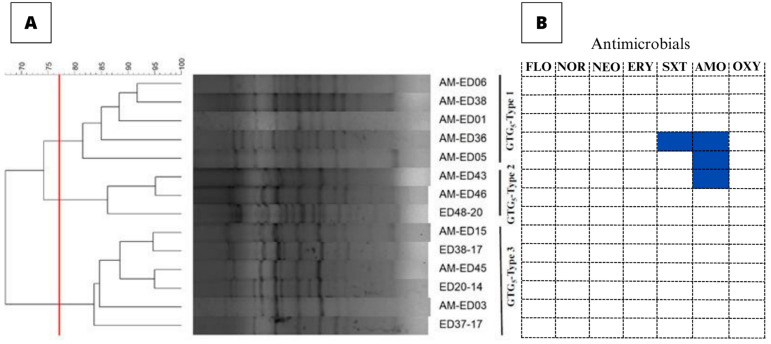
Genetic diversity and antimicrobial susceptibility of *E. tarda* isolates recovered from *C. macropomum*. (**A**): Rep-PCR results for the 14 *E. tarda* isolates isolated from *C. macropomum*. The dendrogram was constructed using the Dice coefficient and the UPGMA method. The red line represents the cutoff defined considering the mean value of all clusters minus 1645 per standard deviation. (**B**): Antimicrobial susceptibility of *C. macropomum*-derived *E. tarda* isolates. Blue boxes represent NWT isolates.

Rep-PCR of the 14 *E. tarda* isolates resulted in the amplification of 8–15 bands, the sizes of which ranged from 400–4000 bp. Three different GTG5 patterns were detected based on a calculated similarity cutoff of 77.08% (Figure 2A). No association was observed between the GTG5-type and the geographic origin of the isolates. The rep-PCR had a discriminatory power of 0.692.

### 3.3. Antimicrobial Susceptibility

The inhibition zone diameter values obtained for the quality control isolates (*Escherichia coli* and *Aeromonas salmonicida* subsp. *salmonicida*) were within acceptable ranges as determined by the CLSI. Quality control ranges for NOR, NEO, and AMO were not yet established, hence inhibition zone diameter could not be compared. *E. tarda* isolates exhibited inhibition zones the diameters of which ranged from 6 mm (disc diameter) to 45 mm. The CO_WT_ values and the inhibition zone diameters are presented in Table S1. All 14 *E. tarda* isolates were classified as WT to FLO, NOR, NEO, ERY, and OXY. Nevertheless, 7.1% and 21.4% of the isolates were classified as NWT to SXT and AMO, respectively (Figure 2B). None of the isolates presented multiple-drug reduced susceptibility.

### 3.4. Challenge Assay

**Figure 3:**
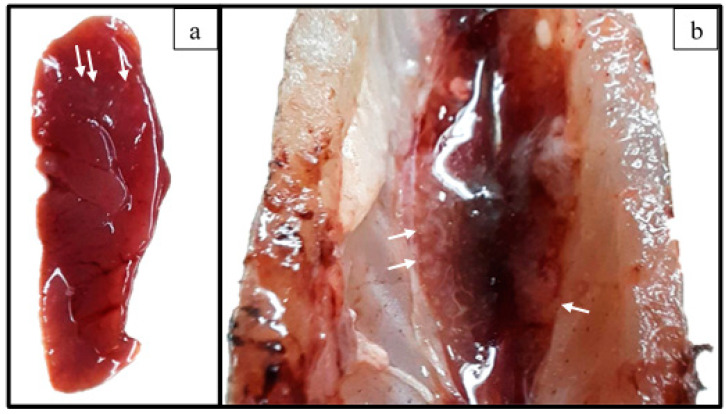
Photographs of the spleen (**a**) and kidneys (**b**) of a *C. macropomum* juvenile experimentally infected with *E. tarda* ED38-17, showing splenomegaly, as evidenced by the round edges of the organ (**a**), and the small, whitish, and round-shaped areas (arrows) in the spleen and kidney (**b**).

Feed intake did not occur during the first day post-inoculation (dpi) in either the challenged or non-challenged group. However, on the second dpi and afterward, the feed intake of the animals in both groups was normal, as observed during the acclimation phase. All fish from both treatment groups survived throughout the experimental period without the development of clinical signs. Euthanasia of all animals was performed at 21 dpi, followed by necropsy. During inspection of the internal organs, splenomegaly and small, whitish, and round-shaped areas in both the kidney and spleen of two challenged tambaqui juveniles were observed (Figure 3). Bacteriological examination of these challenged animals revealed that *E. tarda* was recovered from the internal organs of five fish (83.34%). These organs were the spleen (3/6), liver (3/6), and kidney (1/6). Bacterial recovery was not observed from non-challenged fish. Ultimately, the results of the challenge assay satisfied Koch’s postulate.

### 3.5. Histological Examination

**Figure 4:**
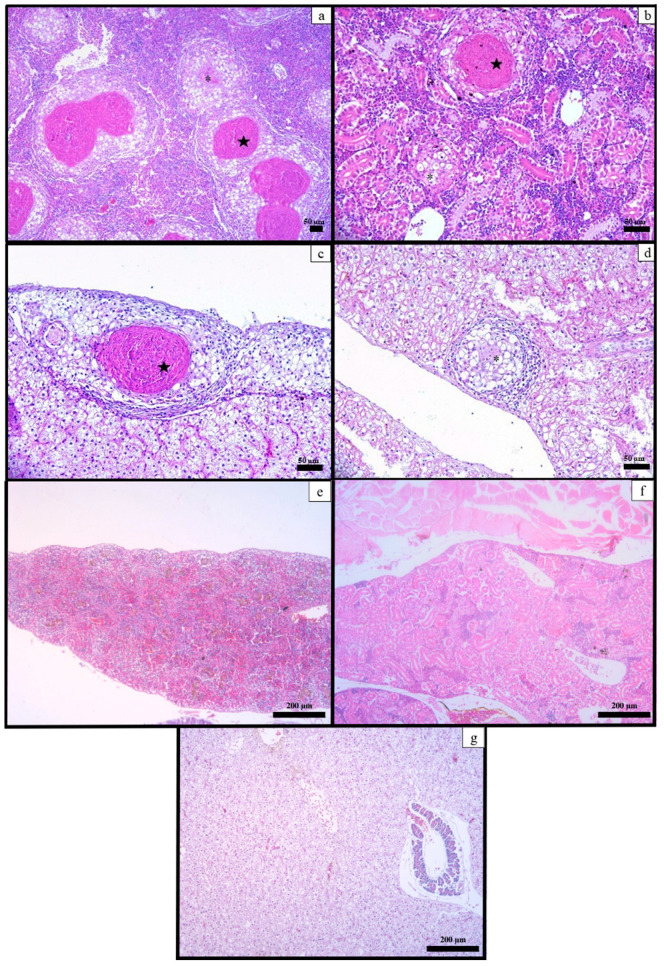
Microphotographs of HE staining of the organs from *C. macropomum* juveniles. Fish experimentally infected with *E. tarda* ED38-17 exhibited granulomas, both with a necrotic center (star) and without a necrotic center (asterisk), in the spleen (**a**), kidney (**b**), and liver (**c**,**d**). Spleen (**e**), kidney (**f**) and liver (**g**) from control group fish are depicted without alterations.

Upon histological examination, the non-challenged tambaqui exhibited typical histological features of healthy fish. However, the *E. tarda*-challenged fish developed several granulomas, mainly composed of epithelioid macrophages and rare lymphocytes. Some granulomas also had necrotic centers. These histopathological alterations were found in the spleen (5/6 fish), liver (5/6 fish), and kidney (1/6 fish), as shown in Figure 4.

## 4. Discussion

*E. tarda* is a bacterial pathogen that affects a broad range of fish species [17]. Although previous reports have described the isolation of this pathogen from tambaqui, a clear association with edwardsiellosis could not be found [16]. The higher occurrence of *E. tarda* in tambaqui over the years reported here is a threat to aquaculture; hence, this subject needs to be further investigated to better understand and address this problem. The missing primordial information was the susceptibility of *C. macropomum* to *E. tarda*. Once this was confirmed, the genetic diversity and antimicrobial susceptibility profiles of different *E. tarda* isolates recovered from tambaqui became relevant to the better comprehension and control of these organisms.

The first isolation of *E. tarda* from tambaqui collected from Aquavet and LAMAO occurred in 2014. Over the years, the occurrence of this bacterium in tambaqui has increased and, in a few cases, clinical signs were also present. *E. tarda*-infected fish commonly showed coelomic distension, exophthalmia, rectal prolapse, petechial hemorrhages, swollen internal organs, and granulomas, which were not observed in the cases described here. However, the clinical signs of *E. tarda* infection in fish may vary after onset [10,17]. 

During bacteriological examination, *E. tarda* was isolated from the kidney, spleen, liver, brain, and intestines of *C. macropomum*. In other fish species, *E. tarda* has been isolated from a broad range of organs, including the organs where *E. tarda* was found in tambaqui [12,15,35,36,37]. The identification of the *E. tarda* isolates was conducted using MALDI-TOF MS followed by confirmation through *dnaJ* gene sequencing [9,38]. The combination of these methods ensured the precision of the identification. This is important, since it has been reported that *E. anguillarum* and *E. piscicida* were mistakenly identified as *E. tarda*. Additionally, an *E. tarda*-specific peptide peak was also identified [9].

After challenging *C. macropomum* with *E. tarda*, no clinical signs or death were detected, whereas bacterial recovery and histopathological alterations were observed. On the other hand, when *E. tarda* in similar bacterial doses were experimentally injected in other fish species, both clinical signs and mortality were observed [35,39,40]. *E. tarda* is known to induce strong clinical signs and high mortality rates (56–90%) in other fish species [35,41,42]; however, *E. tarda* has been isolated from clinically healthy tambaqui [16]. The differences in clinical status may be related to the characteristics of the isolate or the host, or the presence of predisposing factors. For example, the age of the fish can potentially modulate the results, producing clinical signs or mortality, but further investigations are needed. The use of an *E. tarda* isolate retrieved from a tambaqui without clinical signs is a limitation of this study. An *E. tarda* infection with clinical manifestation would result in more precise outcomes. Moreover, a longer observation period after challenge may be necessary for tambaqui to display signs of disease, since granulomas, the main histopathological lesion found in this fish, are usually related to chronic infections. 

*E. tarda*-infected tambaqui showed macroscopic white nodules on the kidney, spleen, and liver, which correlated with the histopathological findings, indicating the establishment of multifocal granulomatous inflammation. Granulomas were also observed in tilapia (*Oreochromis* spp.) and Japanese snapper (*Pagrus major*) [43,44], but these differed from the histological lesions found in the Japanese flounder (*Paralichthys olivaceus*; suppurative inflammation in the kidney and liver), channel catfish (*Ictalurus punctatus*; necrotizing inflammation in the kidney, liver, and spleen), and seahorse (*Hipocampus erectus*; enteritis) [35,43,45]. Although the exact cause of granulomas is still unknown; granuloma is the product of a complex process involving the defense mechanisms of the host used to eliminate the pathogen and the strategies of evasion of the bacteria used to resist the immunity [46]. 

Once the pathogenicity of *E. tarda* in tambaqui was demonstrated, a rep-PCR analysis was conducted, in which the *C. macropomum*-derived *E. tarda* isolates were clustered into three distinct groups, indicating that they were not clonally related. Similar findings were observed in studies analyzing *E. tarda* isolates derived from a single fish species [47] and from multiple fish species [48], in which two distinct *E. tarda* groups were found on both occasions. The greater number of genetic variants found among the tambaqui-derived *E. tarda* isolates may be the outcome of a stress adaptation process triggered by the need to survive in a new host [49]. 

An antimicrobial susceptibility test was also conducted on *C. macropomum*-derived *E. tarda* isolates. The NRI method was employed for statistical validation and standardization for the establishment of the epidemiological cutoff values, resulting in an increase in the accuracy of the resistance data. However, since few isolates were analyzed, the isolates were infecting a new host, and they showed great genetic diversity, possibly leading to different antimicrobial susceptibility patterns from those previously described for other isolates, cutoff values must be considered as only provisional. Also, the antimicrobial susceptibility test data should not be used as a basis for clinical recommendations, since some of these drugs are not approved for use in aquaculture. This analysis revealed that all of the isolates were WT to all antimicrobial agents tested, except for AM-ED36 to SXT and AM-ED05, AM-ED36 and AM-ED43 to AMO. Interestingly, similar cutoff values (Table S1) were found for 52 *E. tarda* isolates mainly recovered from farmed eels and olive flounder in South Korea [50], which may be related to the use of NRI in both studies. Other studies have shown different levels of agreement with our antimicrobial susceptibility findings [12,51,52,53,54,55,56]. The susceptibility of these isolates to OXY and FLO is of great importance, since these are the only drugs allowed to be used in Brazilian fish farms. However, since *E. tarda* was not known to infect *C. macropomum*, no drug has been ever applied in in vivo studies for the treatment of edwardsiellosis in tambaqui.

## 5. Conclusions

In conclusion, the data presented here show that *E. tarda* ED38-17 was able to infect and induce tissue lesions in *C. macropomum*, potentially threatening the production of this Amazonian fish species. The *E. tarda* isolates recovered from *C. macropomum* have three distinct genetic types and are WT to FLO, NOR, NEO, ERY, and OXY. 

## Figures and Tables

**Table 1:** Metadata of the *Colossoma macropomum*-derived *Edwardsiella tarda* isolates.

Isolate	Culture Collection	Clinical Status of the Fish	State	Year of Isolation	Organ	MALDI-TOF Score Value
ED20-14	Aquavet	Diseased	MG	2014	Kidney	2.494
ED37-17	Aquavet	Healthy	RO	2017	Kidney	2.358
ED38-17	Aquavet	Healthy	RO	2017	Kidney	2.534
AM-ED01	LAMAO	Healthy	AM	2018	Brain	2.210
AM-ED03	LAMAO	Healthy	AM	2018	Brain	2.459
AM-ED05	LAMAO	Diseased	AM	2019	Kidney	2.153
AM-ED06	LAMAO	Healthy	AM	2019	Brain	2.598
AM-ED15	LAMAO	Diseased	AM	2019	Brain	2.521
AM-ED36	LAMAO	Healthy	AM	2019	Spleen	2.539
AM-ED38	LAMAO	Diseased	AM	2019	Kidney	2.600
AM-ED43	LAMAO	Diseased	AM	2019	Liver	2.286
AM-ED45	LAMAO	Healthy	AM	2019	Intestines	2.292
AM-ED46	LAMAO	Healthy	AM	2020	Kidney	2.347
ED48-20	Aquavet	Healthy	AM	2020	Intestines	2.316

## Data Availability

*DnaJ* gene sequences from *E. tarda* isolated from *C. macropomum* were included in the NCBI database as follows: AM-ED01: OP535031; AM-ED03: OP535032; AM-ED05: OP535033; AM-ED06): OP535034; AM-ED15: OP535035; AM-ED36: OP535036; AM-ED38: OP535037; AM-ED43: OP535038; AM-ED45: OP535039; AM-ED46: OP535040; ED20: OP535041; ED37: OP535042; ED38: OP535043; ED48: OP535044.

## Supplementary Materials

The following supporting information can be downloaded at: https://www.mdpi.com/article/10.3390/ani13182910/s1, Table S1: Antimicrobial susceptibility patterns of the *E. tarda* isolates. The average inhibition zone diameters (mm) and the provisional epidemiological cutoff (CO_WT_) values (mm) of each isolate are presented. Isolates classified as NWT for a certain antibiotic are highlighted in gray-colored cells.
