# Supplementary material for: Edwardsiella tarda in Tambaqui (Colossoma macropomum): A Pathogenicity, Antimicrobial Susceptibility, and Genetic Analysis of Brazilian Isolates
Source: Animals (Basel). 2023 Sep 14;13(18):2910. doi: 10.3390/ani13182910 (PMC10526069; doi:10.3390/ani13182910)
Supplement: Supplementary file 1 [file animals-13-02910-s001.zip › animals-2590471-supplementary.pdf]

Table S1. Antimicrobial susceptibility patterns of the *E. tarda* isolates. The average inhibition zone diameters (mm) and the provisional epidemiological cutoff (CO_{WT}) values (mm) of each isolate are presented. Isolates classified as non-wild-type for a certain antibiotic are highlighted in gray colored cells.

		Antimicrobial agent						
		FLO	NOR	NEO	ERY	SXT	AMO	OXY
Colossoma macropomum	AM-ED01	31	26	11	12	23	27	28
	AM-ED03	33	37	12	11	31	28	32
	AM-ED05	32	34	12	13	25	6	29
	AM-ED06	33	35	11	14	24	28	27
	AM-ED15	33	36	11	13	26	27	27
	AM-ED36	32	27	12	12	6	6	28
	AM-ED38	34	33	12	13	26	27	28
	AM-ED43	32	30	11	13	27	6	29
	AM-ED45	36	36	12	13	32	26	34
	AM-ED46	33	34	12	12	28	26	29
	ED20-14	33	36	11	12	26	28	29
	ED37-17	33	35	13	15	31	30	32
	ED38-17	32	29	12	12	25	27	27
	ED48-20	32	36	12	14	26	27	28
	**CO_{WT} value**	**27**	**26**	**10**	**8**	**18**	**23**	**25**
Oreochromis niloticus	ED51-20	35	24	15	12	34	27	6
	AM-ED08	35	38	11	12	24	29	28
Arapaima gigas	AM-ED10	32	32	11	11	27	27	28
	AM-ED24	35	36	11	12	27	26	29
Brycon amazonicus	AM-ED34	35	36	12	11	27	27	31
Symphysodon ssp.	AM-ED04	33	36	11	13	26	29	30
Pterophyllum scalare	AM-ED47	33	34	11	11	30	28	30
	ATCC25922	23	33	15	13	26	16	25
	ATCC33658	33	34	16	23	26	33	29

FLO, florfenicol; NOR, norfloxacin; NEO, neomycin; ERY, erythromycin; SXT, sulfamethoxazole-trimethoprim; AMO, amoxicillin; OXY, oxytetracycline.

ATCC25922 and ATCC33658 are, respectively, *Escherichia coli* and *Aeromonas salmonicida* subsp. *salmonicida* used as control. CLSI QC ranges for NOR, NEO and AMO are not yet established.
